# Clinical Applications of Antisense Oligonucleotides in Cancer: A Focus on Glioblastoma

**DOI:** 10.3390/cells13221869

**Published:** 2024-11-11

**Authors:** Alexandre Khuu, Maïté Verreault, Philippe Colin, Helene Tran, Ahmed Idbaih

**Affiliations:** 1 AP-HP, Institut du Cerveau, Paris Brain Institute, ICM, Inserm U 1127, CNRS UMR 7225, Hôpitaux Universitaires La Pitié Salpêtrière, Charles Foix, DMU Neurosciences, Service de Neuro-Oncologie-Institut de Neurologie, Sorbonne Université, 75013 Paris, France; alexandre.khuu@servier.com (A.K.); maite.verreault@icm-institute.org (M.V.); 2 Institut de Recherche Servier, Rue Francis Perrin, 91190 Gif-sur-Yvette, France; philippe.colin@servier.com

**Keywords:** antisense oligonucleotides, oncology, clinical applications, glioblastoma

## Abstract

Antisense oligonucleotides (ASOs) are promising drugs capable of modulating the protein expression of virtually any target with high specificity and high affinity through complementary base pairing. However, this requires a deep understanding of the target sequence and significant effort in designing the correct complementary drug. In addition, ASOs have been demonstrated to be well tolerated during their clinical use. Indeed, they are already used in many diseases due to pathogenic RNAs of known sequences and in several neurodegenerative diseases and metabolic diseases, for which they were given marketing authorizations (MAs) in Europe and the United States. Their use in oncology is gaining momentum with several identified targets, promising preclinical and clinical results, and recent market authorizations in the US. However, many challenges remain for their clinical use in cancer. It seems necessary to take a step back and review our knowledge of ASOs and their therapeutic uses in oncology. The objectives of this review are (i) to summarize the current state of the art of ASOs; (ii) to discuss the therapeutic use of ASOs in cancer; and (iii) to focus on ASO usage in glioblastoma, the challenges, and the perspective ahead.

## 1. Introduction

In recent years, biomedical research has highlighted the crucial role of messenger ribonucleic acids (mRNAs) in the pathophysiology of infectious, neurological, or oncological diseases. These pathogenic mRNAs are now major therapeutic targets in diseases previously considered incurable [1,2,3]. Indeed, mRNAs have the advantage of being sufficiently upstream in the pathophysiology of diseases and being sufficiently far away from the genome to avoid any mutagenic risk. Developing a new technology capable of targeting these pathogenic mRNAs has required considerable effort from the scientific community. The pioneering work by Stephenson and Zamecnik in 1978, which demonstrated the feasibility of targeting pathogenic mRNAs for therapeutic purposes, marks the birth of what would later be termed antisense oligonucleotides (ASOs) [4,5]. These new therapeutic entities located between chemistry and biology represent a promising therapeutic alternative thanks to the many advantages they can offer. Indeed, they can modulate the expression of virtually any target RNA, and the drug design is flexible and streamlined [6,7]. In addition, the sequence of their targets can be easily obtained using next-generation sequencing (NGS) techniques. Their production is relatively inexpensive thanks to production automation and the components’ availability [8]. Unfortunately, ASOs have a risk of causing adverse effects related to off-target hybridizations [9,10]. Since their discovery, several waves of preclinical and clinical studies have made it possible to optimize their pharmacological, pharmacodynamic, and pharmacokinetic properties; increase their field of use; and decrease their overall toxicities [11,12,13,14]. They are mainly used in neurology and metabolic diseases, and their use in oncology is gaining momentum with the recent approval of imetelstat by the FDA for low- to intermediate-1-risk myelodysplastic syndromes with transfusion-dependent anemia. Furthermore, several targets are continuously being explored [15,16,17]. Despite those advances, there are still many challenges to overcome before they can be used more widely against tumors: (i) the choice of the target; (ii) their ability to cross biological barriers; (iii) their biodistribution in the tumor; (iv) their degradation by endo- and exonucleases present ubiquitously in the body; and (v) their heterogeneous productive cellular uptake [18,19,20,21,22]. This review aims to summarize existing knowledge on ASOs and provide insight into their current clinical applications in oncology with a specific focus on glioblastoma.

## 2. Antisense Oligonucleotides: The State of the Art

### 2.1. The Family of Therapeutic Oligonucleotides

**Figure 1:**
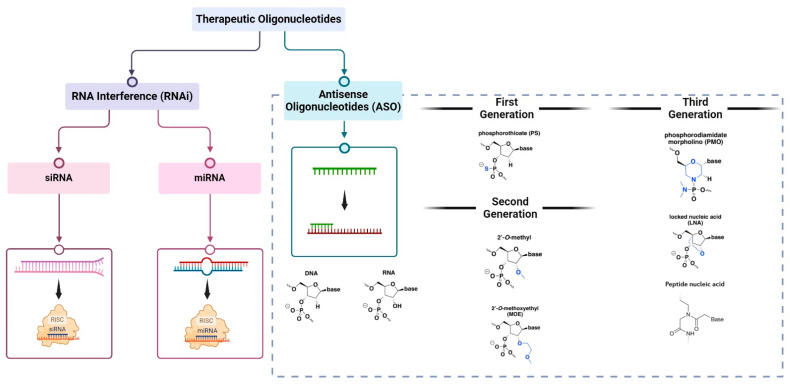
Therapeutic oligonucleotides modalities: RNA interference (RNAi) and antisense oligonucleotides (ASOs). ASOs can be chemically modified, and the most investigated modifications per generation are described here.

Therapeutic oligonucleotides are synthetic nucleic acids divided into two main classes: (i) antisense oligonucleotides (ASOs) and (ii) RNA interference (RNAi), which are subdivided into small interfering RNA (siRNA) and microRNA (miRNA) [23] (Figure 1). Their main difference resides in their structures. ASOs are single-stranded with 10 to 30 mers while RNAis are double-stranded with 20 to 25 mers. This difference in structure has consequences on the mechanisms of action and the physicochemical ability of each class. Thus, ASOs can be less stable but will singly engage their targets. In contrast, RNAis can be more stable but will require binding with intracellular Argonaut proteins (AGO) to form an RNA-induced silencing complex (RISC) to engage their targets. This binding causes a separation of the two strands into one active passenger strand complementary to the target, and one guide strand helping with cellular machinery recognition. This effectively makes RNAis prodrugs because they require a transformation step to be active. However, in this review, we will only focus on ASOs.

As nucleic acids, ASOs have sequences that can hybridize their RNA targets (pre-mRNA, mRNA, tRNA, or miRNA) by complementary base pairing to exert their pharmacological effects. The presence of this sequence represents a paradigm shift with conventional small molecules. For the latter, their chemical structure determines both their pharmacokinetic properties (dianophore) and their pharmacodynamic properties (pharmacophore). In the case of oligonucleotides, we enter the field of what Khvorova et al. called informative drugs, where the dianophore depends on the chemical structure, while the pharmacophore depends on the sequence [24]. This separation makes it possible to optimize the dianophore independently of the pharmacophore, offering development flexibility that is unprecedented in drug development. Indeed, it is possible to design a sequence for a specific target and then create different chemical structures for each tissue where the target would be located. Conversely, one can develop a chemical structure for a particular tissue and then adapt the sequence for the different targets present in that tissue [25].

Despite this developmental flexibility, some parameters have been associated with better ASO efficiency, even if there are no absolute rules in terms of design [26]: (i) the secondary structure of the target mRNA strongly influences its accessibility and the hybridization of the ASOs [27,28]; (ii) the secondary structure of the ASOs can influence the quality of hybridization [29,30]; (iii) in the ASOs sequence, the abundance of GC nitrogenous bases (guanine and cytosine) and certain sequence patterns increase ASO activity by increasing the stability of the target ASO/RNA complex [31]; and (iv) the thermodynamic binding energy of ASOs must be taken into account because the affinity of ASOs must be more favorable to hybridization between ASOs and their target mRNAs as opposed to hybridization with other entities, such as other ASOs or proteins [32]. Many tools have been developed over the years to assess and predict those parameters [32,33,34].

### 2.2. Pharmacological Properties of Antisense Oligonucleotides

ASOs have a DNA- or RNA-based structure with chemical modifications on their sugars, phosphodiester bonds, and nitrogenous bases to improve their physicochemical, pharmacokinetic, and pharmacodynamic properties. These modifications are used to classify them into distinct chemical classes with different pharmacological properties. To date, three generations of ASOs have been developed, each aimed to improve ASOs pharmacokinetics, efficacy, tolerability, and immunogenicity [12,35,36,37,38] (Table 1).

The first generation of ASOs is centered around the modifications of the phosphates involved in phosphodiester bonds. One of the oxygen atoms (O) of these bonds can be replaced either by a sulfur atom (S) (ASO-PS), a methyl group (CH3) (ASO methyl-phosphonate), or an amine group (NH) (ASO phosphorodiamidate). Of all these changes, ASO-PS is the most widely used today [39]. The second generation of ASO was developed in part to correct the toxicity of the first generation and is characterized by the modification of the hydroxyl group (OH) in position 2′ of the ribose. This group can be replaced by a methyl group (ASO-2′-O-Me) or by a methoxyethyl group (ASO-2′-MOE) [40]. This generation also comprises gapmers, ASOs where the central parts have a different chemical scaffold than its 5′ and 3′ ends to combine different advantages of varying chemical classes [41]. The third and most recent generation of ASOs is characterized by complete changes in the nucleotide structure. The most investigated include (i) ASO-LNA (ASOs with a bridged ribose) [42]; (ii) ASO-PMO (ASOs where the ribose has been replaced by a morpholine group and the phosphate of the phosphodiester bond has been replaced by a phosphorodiamidate group) [43] and; (iii) ASO-PNA (ASOs where the ribose and phosphates have been replaced by amino acids) [44].

### 2.3. Pharmacodynamic

**Figure 2:**
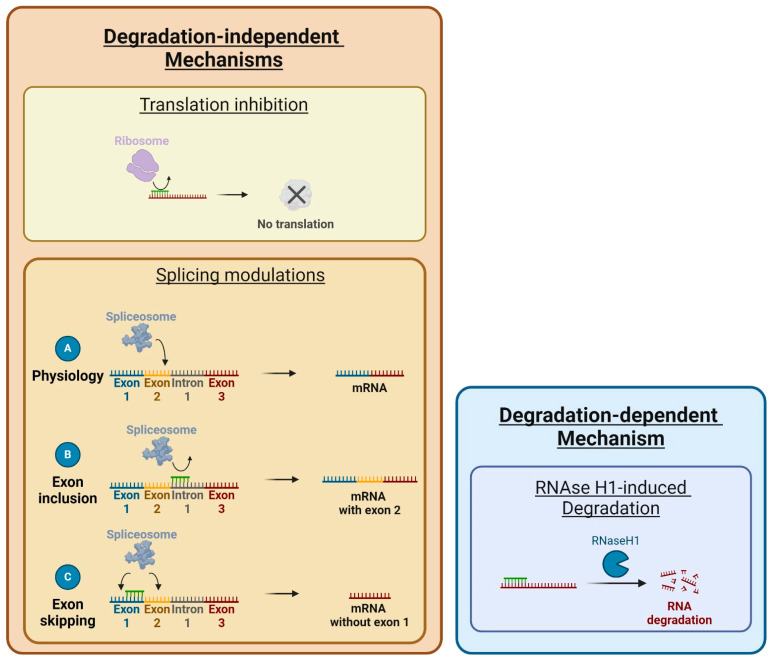
Mechanism of action of ASOs. (i) Degradation by RNase H1. After hybridization of the ASO to its RNA target, RNase H1 binds to the duplex and begins cleavage. The scissors indicate the sites of possible cleavage. (ii) Inhibition of translation. After hybridization of the ASO to its mRNA target, the ASO blocks the binding of the ribosome. (iii) Splicing modulations. Splicing factors exist to promote or inhibit splicing sites. With ASOs, we can either hide the binding sites of the promoters and cause exon skipping or the binding sites of inhibitors and cause exon inclusion.

To exert their pharmacological effects, ASOs must hybridize with their RNA targets, whether in the nucleus or the cytoplasm. The proteins involved, such as chaperone proteins and regulatory proteins, along with the selection of the target sequence, are critical and have been the focus of extensive research [45,46]. The most relevant region is important for the mechanism of action exerted by the ASO and must have a minimum size of 13 bases. Indeed, starting from 13 bases, we can consider that the sequence is statistically unique in the human genome and therefore specific to a defined target [47]. Once hybridized, the mechanisms of action of ASOs can be divided into two broad categories: (i) degradation-dependent mechanisms and (ii) degradation-independent mechanisms (Figure 2).

Degradation-dependent mechanisms are the most used mechanism among marketed ASOs and mainly concern degradation by RNase H1, a ubiquitous enzyme of DNA replication in eukaryotic cells [37,48,49]. For example, it is the mechanism used by the ASO volanesorsen authorized in the European Union to treat familial chylomicronemia syndrome. This ASO degrades the mRNA of apolipoprotein C-III, preventing its inhibition of triglyceride metabolism [50]. RNase H1 cleaves the RNA-ASO duplexes formed after hybridization of at least 6-7 bases, degrading the RNA and leaving the ASO intact [51]. RNase H1 binds to RNA-ASO complexes via a hybrid binding domain (HBD) located at the N-terminal end of the enzyme. The HBD domain must recognize both an RNA strand and a DNA strand to activate the enzyme [48]. Therefore, ASOs must have a DNA-based structure to be recognized by RNase H1 [52]. Depending on the target sequence, structural conformations can vary, which influences the binding of RNase H1 to RNA–ASO duplexes. These sequences have yet to be determined, but some have already been identified, such as the CAAG sequence downstream of a G-rich sequence.

Degradation-independent mechanisms are more diverse and include the mechanisms of translation inhibition and splicing modulation [37]. These mechanisms include ASOs with no DNA-based structure, which are thus incapable of recruiting RNAse H1, such as ASO-PNA, ASO-PMO, ASO-2′-O-Me, and ASO-2′-MOE (without gapmer). For translation inhibition, the ASOs bind to their target without inducing their degradation. They create a steric blockage on the mRNAs, inhibiting ribosome binding during translation. Some sequences are preferred as targets, particularly the translation initiation codon (AUG) or sequences close to the latter [26]. No commercially available ASOs are using this mechanism yet. However, two Phase 1 clinical trials used this mechanism to knock down the proto-oncogene *c-MYC*, which is overexpressed in many cancers [53]. AVI-4126 is a 20-mer ASO-PMO developed by Sarepta Therapeutics [54]. It prevents the translation of the c-Myc protein by steric blockade, resulting in cellular growth inhibition in vitro and in vivo. The results of two Phase 1 studies on breast and prostate cancer patients and healthy subjects show good pharmacokinetic and toxicity profiles for this ASO. However, no efficacy data on the target were generated.

For the splicing modulation, they can modulate the splicing of pre-mRNAs. ASOs have two effects depending on their binding site, they cause either an exon skipping or an exon inclusion. For exon skipping, the ASO binds to a site promoting splicing of the pre-mRNA and shifts the reading frame, resulting in a shorter mRNA [55]. It is the mechanism of many ASOs authorized by the US FDA to treat Duchenne muscular dystrophy (DMD). These ASOs bind to the pre-mRNA of the *DMD* gene to exclude exon 51, 53, or 45 to restore a shorter but functional dystrophin protein [56]. For exon inclusion, ASO binds to a site inhibiting splicing of the pre-mRNA, preventing splicing inhibitors from accessing their binding site, resulting in a longer mRNA [57]. It is the mechanism of ASO nusinersen, which is authorized in the European Union to treat spinal muscular atrophy (SMA). It binds to the pre-mRNA of the gene Survival of Motor Neuron 2 (*SMN2*) and modulate its splicing to include an exon necessary to produce the SMN protein [58]. The targeted sequence for both exon inclusion and exon skipping is strongly linked to the sequences used by the splicing promoter and inhibitor proteins [26].

### 2.4. Pharmacokinetic

The pharmacokinetic parameters influencing absorption, distribution, metabolism, and elimination of naked ASOs in humans, i.e., without formulation, vary for each chemical class of ASO, of which the first and second generations are best described. Studies on the third generation are still limited to preclinical models.

The use of ASOs by parenteral routes is preferred because they are poorly absorbable orally because of their high molecular weight, hydrophobicity, and electrical charge [59,60,61,62,63].

Once absorbed, ASOs can be free or bound to plasma proteins (mainly albumin and alpha-2-macroglobulin). First- and second-generation ASOs are more than 90% bound to plasma proteins due to their high hydrophobicity [64]. This allows them to have reduced renal clearance, a longer half-life, and good tissue distribution in highly perfused tissue (i.e., the kidneys, liver, and lymphatic system) [65]. Third-generation ASOs bind very little to plasma proteins, especially because some do not have an electrical charge (such as ASO-PNA and ASO-PMO). This results in decreased tissue accumulation, primarily in the kidneys and liver, and reduced bioavailability, with distribution half-lives of approximately ten minutes in rats and mice [66,67,68,69].

The metabolism of ASO is mainly done by endonucleases and/or exonucleases that are ubiquitous in the body, with the first-generation ASOs being the least resistant and the third generation being almost non-metabolized [65,67]. The elimination of ASOs and their metabolites is mainly done through the urine with varying half-lives depending on the generation [66,68,69].

On the cellular level, ASOs without formulation enter the cells in precise steps that are the subject of intense research [70]. When ASOs reach cell membranes from the bloodstream, they can be absorbed by cells. This absorption is done in two stages: (i) an adsorption step and (ii) an internalization step [70,71]. The adsorption is mainly due to the binding of ASOs to membrane proteins [72] such as integrins [73], G protein-coupled receptors (GPCR) [74], or Scavenger receptors [75]. Once adsorbed, the ASOs are internalized by different pathways depending on the membrane protein involved, and endocytosis has been identified as the main one [76]. The mechanisms of endocytosis are numerous, and the best studied remain clathrin-dependent endocytosis and caveolin-dependent endocytosis [77]. Very few of these internalizations result in pharmacological activity [78]. This is called non-productive internalization, as opposed to productive internalization [72,79]. Productive internalization is limited and heterogeneous depending on (i) ASO chemistry, (ii) administration modalities, (iii) cell type, and (iv) adsorption protein [80,81,82].

**Figure 3:**
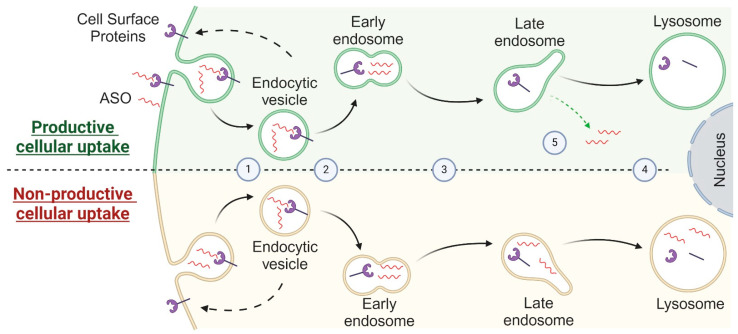
(1) After internalization, the initial endocytic vesicle containing the ASOs fuses with the early endosome. (2) The early endosome, located close to the cell membrane, has an acidic pH between 6 and 6.5. It sorts its contents, separating the ASOs from the proteins. The proteins are either recycled to the plasma membrane or continue their journey with the ASOs to the late endosome. (3) The late endosome also serves as a sorting platform, directing its content to other cell structures or lysosomes. Proteins are directed to the lysosome for degradation. (4) The lysosomes are characterized by an acidic pH between 4.5 and 5.5 and are rich in hydrolases that facilitate the breakdown of proteins, nucleic acids, and ASOs. In the case of non-productive internalization, the ASOs complete their journey in the lysosomes. (5) Conversely, in cases of productive internalization, ASOs escape the late endosome through various mechanisms. These mechanisms involve temporary and spontaneous permeability of the late endosome during intracellular trafficking.

After internalization within the cell, ASOs are transported by the cellular machinery through different compartments in a process called intracellular trafficking [83,84,85,86,87,88] (Figure 3).

Interestingly, this pharmacokinetic profile is quite slow and time-consuming [45]. For instance, RNAse H1-induced degradation takes on average two hours between the absorption of ASOs by the cell and its action on the target. The ASO takes an hour to be absorbed and to make its way to its target. It then takes an extra hour to degrade it. Finally, ASOs can be recycled by being driven back to a new target or excreted by the cell in exosomes to start a new cellular absorption process in other cells [89].

### 2.5. Toxicity and Adverse Effects

Like every other drug, ASOs have some toxicity causing different side effects [13]. These adverse reactions can be divided into two categories: (i) hybridization-dependent effects or (ii) hybridization-independent effects.

Hybridization-dependent effects are caused by the hybridization of ASOs to their target. They can be an exacerbation of the pharmacological effect or an off-target effect if there is total or partial hybridization to an unexpected target [30]. Total hybridizations are rare, so partial hybridizations are more common and can still recruit RNase H1 [90]. These partial hybridizations can be predicted by in silico analyses that will determine the number of potential unexpected targets [49]. The number of unexpected targets is proportional to the number of non-corresponding bases during the hybridization of ASOs and their targets [91,92]. In addition, the off-target effects of ASOs with degradation-independent mechanisms of action are more numerous. Indeed, they can act as miRNAs and require fewer hybridized bases than an ASO with a degradation-dependent mechanism to cause a pharmacological effect [93].

Hybridization-independent effects are caused by all other ASO interactions that are not hybridizations [30]. These effects are generally dependent on chemistry and sequence, but we will look at the most common effects such as pro-inflammatory effects. These effects are mainly due to the recognition of ASOs by receptors of pathogen-associated patterns (PAMPs) such as receptors of the Toll-Like family (TLR) and in particular TLR9 [94,95]. In rodents, cytokine release, lymphoid hyperplasia, and lymphocyte infiltration have been observed [96]. In primates, vasculitis and glomerulonephritis linked to complement system activation have been observed [97]. In the clinic, flu-like syndrome and inflammatory reactions at the injection site have been observed [98]. Other common effects are the effects of liver and kidney toxicities. Liver toxicity can manifest as both inflammatory and non-inflammatory reactions, leading to hepatocyte death in rodents, primates, and humans [99,100]. The mechanisms invoked are aptameric interactions with hepatic intracellular proteins [101]. Renal toxicity is caused by the accumulation of ASOs in the lysosomes of proximal tubule cells, leading to increased proteinuria and tissue necrosis [102]. Another common hybridization-independent side effect is thrombocytopenia, observed in rodents and primates at high doses [102,103]. Various mechanisms have been proposed but not demonstrated due to a lack of relevant data. These include unintended activation of adenosine diphosphate (ADP) or platelet factor (PF4), key components of platelet activation [104].

Amongst the ASOs that made it to the clinic, their adverse reactions are generally dose-dependent and can therefore be managed according to the therapeutic index of the ASOs [105].

## 3. Antisense Oligonucleotide Therapies in Cancers

### 3.1. A Promising Rationale

Recent advances in genomics have made it possible to define the genes involved in the mechanisms of oncogenesis more precisely [106]. Some of these genes have become therapeutic targets of interest for antisense approaches. Indeed, ASOs can be used against any target RNA through mindful drug design and development flexibility, especially for therapeutic targets that are traditionally difficult to treat. The currently preferred target genes are overexpressed driver genes in cancers such as anti-apoptotic, pro-proliferative, and pro-angiogenic genes that are non-actionable by other treatments [39,107,108,109]. It is interesting to note that ASOs appear to be better absorbed by fast-growing cells such as tumor cells [70]. The use of ASOs in cancers is the subject of numerous research that will not be covered in this review. We found it more relevant to instead focus our review on some ASOs that made it to human administration, noting that most ASOs are in Phase 1/2 clinical trials, some are in Phase 3, and one has received FDA approval [16,110].

### 3.2. Hematological Malignancies

B-cell lymphoma 2 (BCL2) is a mitochondrial transmembrane protein that contributes to the maintenance of mitochondrial membrane integrity [111]. An altered BCL2 protein can promote the stabilization of the mitochondrial membrane and cause cellular escape from the mechanisms of apoptosis [112]. BCL2 is activated in hematological malignancies, such as chronic lymphocytic leukemias, multiple myelomas, and non-Hodgkin lymphomas. In these cases, the therapeutic strategy is to reduce the amount of impaired BCL2 protein. For this, the ASO oblimersen was developed by Genta. It is an 18-mer ASO-PS complementary to the first six codons of BCL2 mRNA that causes its degradation by RNase H1 after its hybridization [113]. Phase 1/2 trials of oblimersen have been promising. Indeed, oblimersen is well tolerated and causes a reduction in BCL2 protein levels after intravenous injection in patients with non-Hodgkin lymphoma [114]. However, all Phase 3 trials with oblimersen alone or in combination with other chemotherapies (docetaxel, fludarabine, and cytarabine) have been negative in many hematological indications [16]. Indeed, the primary objectives of overall survival and progression-free survival were not met. As a result, the development of the oblimersen was stopped. Phase 1 and 2 trials with other anti-BCL2 ASOs are ongoing, such as ASO BP1002, which does not degrade BCL2 mRNA but blocks its translation (NCT04072458).

Signal transducer and activator of transcription 3 (STAT3) is a signal-transducing and transcription-activating protein [115]. Once activated, STAT3 dimerizes and migrates to the nucleus to act as a transcription factor for many genes involved in cell proliferation, migration, invasion, and survival [116]. Abnormal activity of STAT3 has been observed in many cancers, particularly hematological ones, and has proven difficult to inhibit with existing therapies [90,117].

To reduce the amount of STAT3 protein, Ionis developed danvatirsen (AZD9150). It is a 16-mer long ASO-PS with chemical modifications causing a steric blockage on STAT3 mRNA, preventing its translation [118]. In a Phase 1 clinical trial, patients with diffuse large B-cell lymphoma received danvatirsen intravenously. Danvatirsen was well tolerated and showed efficacy signals with partial responses noted in all treated patients and complete tumor responses observed in a few patients [119]. Phase 2 trials are underway for other indications (e.g., colorectal cancer or lung cancer) (NCT02983578) and in combination with the anti-PDL1 antibody Durvalumab (NCT03421353).

### 3.3. Solid Cancers

Protein kinase C (PKCα) is a cytoplasmic serine/threonine kinase involved in signal transduction during cell differentiation and proliferation [120]. Overexpression of PKCα promotes tumor proliferation and resistance to chemotherapy in many cancers, including breast and ovarian cancers [121]. Aprinocarsen was developed by Ionis to reduce the amount of PKCα protein. It is a 20-mer long ASO-PS that recruits RNase H1. In Phase 1/2 clinical trials, patients with different types of solid cancers were treated with aprinocarsen. Some degree of toxicity was observed with low activity [122]. It was therefore decided to use aprinocarsen in combination with cisplatin and gemcitabine in patients with non-small cell lung cancer in a Phase 2 clinical trial. After intravenous infusion, aprinocarsen in combination with conventional chemotherapy provides a better response rate without significant toxicity [123]. However, a Phase 3 clinical trial combining aprinocarsen with cisplatin and gemcitabine yielded negative results, leading to the discontinuation of aprinocarsen’s development [124,125].

Apolipoprotein J (Clusterin) is a heterodimeric secreted glycoprotein that is considered a marker of cellular senescence. Cytokines, growth factors, and stress-induced agents regulate its expression. Its role is poorly understood, but clusterin is thought to be a cytoprotective and anti-apoptotic chaperone protein induced by stress [126]. It is overexpressed in many metastatic cancers, such as colon, bladder, and liver cancers. Its overexpression increases cell migration and participates in the metastatic process and resistance to chemotherapy [127,128]. To reduce the amount of clusterin, Ionis and OncoGenx have developed the ASO custirsen. It is a 21-mer long gapmer ASO-PS-2′-MOE [129]. Phase 3 clinical trials have been conducted in patients with castration-resistant metastatic prostate cancer. Custirsen was injected intravenously in combination with prednisone and docetaxel or cabazitaxel. However, it did not improve the overall survival and even induced serious adverse events (especially neutropenia) in almost a quarter of patients [14,130].

X-linked apoptosis inhibitor protein (XIAP) can bind caspases involved in all phases of apoptosis to inhibit them directly. Thus, it inhibits the pathways of apoptosis mediated by caspases [131]. However, the XIAP protein is overexpressed in many cancers, such as liver, colon, or thyroid cancers. Indeed, it allows cancer cells to escape apoptosis and participates in resistance to chemotherapy [132]. To reduce the amount of XIAP protein, ASO AEG35156 was developed by Aegera. It is a 19-mer ASO-PS-2′-Ome gapmer that induces RNase H1 degradation of XIAP mRNA after hybridization. It was evaluated in a Phase 2 clinical trial in combination with sorafenib in patients with advanced hepatocarcinoma. Following intravenous infusion, it showed improved response rates and overall survival with minimal adverse effects [133]. However, Aegera has not conducted additional clinical trials or communicated any development news.

### 3.4. First Step Toward Clinical Successes

A recent breakthrough was made with the first FDA-approved ASO in cancer, imetelstat of Geron for low- to intermediate-1-risk myelodysplastic syndromes (MDSs) with transfusion-dependent anemia. MDSs are a group of myeloid diseases characterized by the clonal proliferation of myelodysplastic stem cells in the bone marrow and cytopenia, including anemia. They have a risk of progressing to acute myeloid leukemia. The medical need for these syndromes is high as the current treatments are symptomatic and revolve around correcting the cytopenia with transfusions or different drugs, such as erythropoiesis-stimulating agents. Imetelstat is a 13-mer long thiophosphoramidate ASO coupled with a palmitoyl. This conjugation to a C16 lipid increases the lipophilicity of the ASO and thus increases its cellular internalization, cell retention, and efficiency [134]. The mechanism of action of imetelstat was not previously described as it does not act directly on gene or protein expression. Instead, it prevents chromosome degradation by inhibiting the telomerase [135]. Indeed, imetelstat’s sequence is complementary to the template ncRNA within the telomerase RNA subunit, inhibiting its activity by steric blockade. Telomerase is a ribonucleoprotein responsible for maintaining the length of chromosomal telomeres involved in cell survival. In cancers, this telomerase is overactivated and participates in tumor initiation and survival [136]. A pivotal Phase 3 study (NCT02598661) was performed to demonstrate imetelstat’s efficacy [137]. After intravenous infusion, the imetelstat group had a lower need for red blood cell transfusion compared with the placebo group, and no serious adverse effect was observed.

This first approval demonstrates the feasibility of using ASOs as monotherapy in cancer treatment and underscores the significance and effectiveness of conjugated ASOs in overcoming ASO limitations. We are confident this will generate momentum in the field, further enhancing efforts to deliver ASOs as therapeutics for oncology indications.

## 4. Focus on Antisense Oligonucleotides Used in Glioblastoma

### 4.1. Glioblastoma

The medical need for glioblastoma, the most common and serious primary brain cancer in adults, is unmet with few treatment options in cases of relapse and an unfavorable prognosis. As a result, ASOs have been studied as a potential therapy in glioblastoma. Indeed, they apply to many glioblastoma oncogenes involved in angiogenesis and the apoptosis pathway [138].

Glioblastoma represents almost half of malignant brain tumors with an average incidence of 3/100,000. This incidence is age-dependent, rising from 0.15/100,000 for children to 15/100,000 for patients over 75. The prognosis for glioblastoma is poor with a median overall survival of 12 to 18 months, also with a variation according to age. On average, 5% of patients survive beyond 5 years, but this figure drops to 2% for those over 65 [139]. The clinical signs of glioblastoma vary depending on the location and tumor size. Thus, involvement of the frontal lobe can lead to motor disorders, while that of the temporal lobe can lead to cognitive disorders [140].

Currently, surgery is one of the primary treatment options due to its effectiveness and the necessity of obtaining a tumor sample for diagnostic neuropathological analysis [141]. However, sometimes tumors are located in unresectable parts of the brain where surgical resection would cause serious neurological damage [142]. In these cases, neurosurgical intervention is limited to a diagnostic tumor biopsy without tumor resection. After surgery, the standard medical treatment for glioblastoma remains encephalic radiotherapy accompanied by concomitant and adjuvant chemotherapy based on temozolomide, a cytotoxic alkylating agent [143]. The doses of radiation and temozolomide are adapted according to the clinical condition and age of the patients. In cases of relapse, treatment options are limited. Indeed, decision-making is based on previous treatments, the clinical condition of the patient, as well as patient age [143]. This therapeutic impasse is multifactorial and involves (i) the intrinsic resistance of glioblastoma cells to chemotherapy and (ii) the extrinsic resistance associated with the tumor microenvironment (i.e., BBB limiting the penetration of medical treatments, immunosuppressive microenvironment). These resistances can exist from the outset or appear secondarily.

### 4.2. Antisense Oligonucleotides Used in Glioblastoma

The first ASO to enter clinical trials to treat glioblastoma is Ionis’ aprinocarsen described earlier. As a reminder, it is an ASO-PS that is a 20-mer and complementary to the 3′-UTR of the PKCα mRNA, inducing its degradation by RNase H1 [144]. The PKCα protein is a cytoplasmic serine/threonine kinase involved in cell differentiation and proliferation and deregulated in many cancers including glioblastoma [120]. Preclinical studies have confirmed the value of aprinocarsen in glioblastoma [145]. Mice were xenografted with human glioblastoma U-87 cells, subcutaneously or intracranially. These cells have similarities to human glioblastoma cells, including their mode of proliferation and their PKCα protein levels. Aprinocarsen was injected intraperitoneally daily for 21 or 80 days in these mice. Aprinocarsen was observed to be well tolerated and to increase median survival time through inhibition of tumor growth in subcutaneous and intracranial tumors. In addition, aprinocarsen reduced the amount of PKCα protein, without reducing the amount of its epsilon or zeta isoforms. So-called “scramble” ASOs, i.e., with the same nucleotide composition but in a different order, were used as a control. The intratumor amounts of aprinocarsen and scramble ASO observed were identical, although scramble ASOs did not appear to induce an effect. These promising results have encouraged the use of aprinocarsen in clinical trials. Thus, in Phase 1 clinical trials, aprinocarsen was used in patients in a therapeutic impasse with different forms of cancer (solid or hematological) [146]. Aprinocarsen was injected by continuous intravenous infusion for 21 days. Few serious adverse events have been observed apart from fatigue and thrombocytopenia. In addition, two complete remissions could be observed. This led to the start of Phase 2 clinical trials, during which aprinocarsen was used in patients with recurrent glioblastoma [147]. In these trials, aprinocarsen was injected by continuous intravenous infusion for 21 days. As before, mild, and reversible thrombocytopenia were observed. However, the patient’s condition rapidly deteriorated, and no tumor response could be confirmed. These mixed results led to a halt in the development of aprinocarsen for this indication.

Isarna’s trabedersen is another ASO evaluated in clinical trials dedicated to glioblastoma patients. It is an 18-mer ASO-PS targeting TGF-β2 mRNA and degrading it by RNase H1 [148]. TGF-β is a secreted homo-dimeric protein involved in embryogenesis and tissue homeostasis through its actions in tissue regeneration, the immune system, the extracellular matrix, cell proliferation, and cell differentiation [149]. TGF-βs has three isoforms, β1, β2, and β3, which are difficult to distinguish from each other. Their sequences and structural conformations are highly similar, enabling them to bind to the same receptors and trigger identical signaling pathways. However, differences remain between them, as all three isoforms are crucial for the organism’s proper development [150]. Depending on the type of cancer, TGF-β isoforms can be both pro- and antitumor agents [151]. Physiologically, they act as tumor suppressors based on their antiproliferative action. In certain molecular contexts, they can become oncogenic through their action on proliferation, angiogenesis, invasiveness, metastasis, and immune repression. In the case of glioblastoma, TGF-β1 and β2 appear to be strongly secreted by tumor cells with autocrine action on tumor promotion [152]. They are therefore interesting targets for ASOs. This will be confirmed in the preclinical studies of trabedersen on tumor cells of different types (including glioblastoma) and peripheral blood mononuclear cells derived from patients [148]. These studies have demonstrated three points regarding trabedersen: (i) it decreases the secretion of TGF-β2 protein from tumor cells, (ii) it decreases the proliferation of tumor cells, and (iii) it increases the cytotoxic functions of immune cells. Subsequently, preclinical studies in rabbits and primates were conducted to assess the toxicity of trabedersen [153]. Good tolerance without toxicity was observed in animals during intratumor and intrathecal injections. Based on these results, Phase 1/2 clinical trials have been initiated in patients with recurrent glioblastoma [154]. Trabedersen was infused intratumorally with good tolerance and efficacy. Tumor responses were observed, including two complete tumor responses. These findings were validated in a Phase 2b clinical trial, which showed improved overall survival in patients receiving low doses of trabedersen compared to those given higher doses of trabedersen or treated with temozolomide [60]. However, Phase 3 clinical trials were canceled due to an insufficient number of patients, which put a halt to the development of trabedersen [155]. Another reason was also mentioned the insufficient statistical power of the results due to design flaws in Phases 1 and 2 [156].

Despite their failures, all these studies show good tolerance and hope for tumor response to ASO injections into glioblastoma. In addition, various studies are underway to re-purpose ASOs developed for other forms of cancer for the treatment of glioblastoma [155]. Thus, the previously discussed oblimersen and custirsen can be re-evaluated in the context of glioblastoma despite their negative results in their Phase 3 clinical trials.

### 4.3. The Limits of Antisense Oligonucleotides in Glioblastoma

The use of ASOs in glioblastoma remains disappointing despite promising clinical trial results in other cancers. Indeed, some limitations must be overcome to make them fully usable in glioblastoma [16]. The first limitation is related to preclinical cellular and animal models. Indeed, the cell lines mainly used as a model of glioblastoma are commercial cell lines grown in fetal calf serum and have significant molecular differences from clinical glioblastoma cells. Animal models are also often unrepresentative in terms of the tumor microenvironment. As a result, human targets of ASOs are usually different or absent in these preclinical models [155]. New preclinical models are therefore being studied, such as glioblastoma cells derived from patients or cells/animals genetically modified to present mutations and markers found in human glioblastoma [157].

The second limitation is the presence of the BBB that limits the accessibility of ASOs to tumor cells. Indeed, the BBB can be weakened in glioblastoma, but part of the BBB remains intact [158]. Thus, ASOs cannot diffuse throughout the entire tumor following intravenous injection. To circumvent this obstacle, ultrasound can be employed to permeabilize the BBB, enabling large molecules, such as ASOs, to effectively penetrate the brain [159,160]. Specific routes of administration, including alternatives to the intravenous route, can be used to bypass the BBB, such as direct intratumor injections or convection-enhanced delivery (CED). CED promotes the penetration of ASOs into the tumor and tumoral cells through a pressure gradient and not a concentration gradient like other delivery modalities [161].

The third limitation is related to the distribution of ASOs to tumor cells. Indeed, in addition to the BBB, it is necessary that the ASOs can reach the glioblastoma cells and that they can be internalized by the latter [162]. To achieve this, ASOs can be functionalized to target glioblastoma-specific receptors, promoting their internalization [155]. Studies are still ongoing to assess the feasibility of functionalization using aptamers. Aptamers are oligonucleotides selected using a SELEX method to bind to a ligand or protein receptor [163]. They can improve the internalization of the molecules attached, such as AS1411, which can bind to the nucleolin protein of glioblastoma cells (a surface marker of many cancers). This aptamer demonstrated the ability to be internalized specifically by glioblastoma cells with antitumor activity in in vitro models [164]. ASOs can also be functionalized by peptides capable of penetrating cells, such as the PP75 peptide that increased the internalization and efficacy of a siRNA in in vitro glioblastoma models [165]. Finally, ASOs can be combined with lipids, such as Geron’s imetelstat, evaluated in a Phase 2 clinical trial involving children with recurrent glioblastoma [166]. After intravenous infusion, imetelstat demonstrated an ability to cross the BBB and inhibit its target in tumor cells. Despite its effectiveness in MDSs, in this case, it did not demonstrate clinical efficacy and was found to be too toxic, interrupting the trial.

The fourth limitation is related to the inherent complexity of cancers. Currently, ASOs that have received approval or are under development focus on a single molecular alteration associated with pathophysiology. However, cancers arise from a multitude of diverse targets, and addressing only one of these may be insufficient for effective treatment [167]. An alternative may be to use ASOs in combination and not as monotherapy in these complex pathologies [63].

Finally, the last limitation is related to drug interactions. ASOs are bound to plasma proteins, which is also the case with chemotherapy [168]. Hence, in a combination therapy involving ASOs and chemotherapies, there may be competition for plasma protein binding, altering their pharmacokinetics and efficacy [16]. However, some ASOs have weaker plasma protein bindings (such as ASO-PMO or ASO-PNA) which may favor their combination with other treatments [169].

## 5. Conclusions/Discussion

The full potential of ASOs remains impeded by their poor pharmacokinetics. We have seen that the productive internalization by cells is not yet mastered and that certain biological barriers such as the gastrointestinal barrier or the BBB remain difficult to overcome. To work around these problems, ASOs are subject to galenic optimization such as bio-conjugations and nanoparticles [162]. We have discussed some examples of bioconjugation with imetelstat that aim to facilitate the interaction of ASOs with lipophilic elements, such as cell membranes. Another example includes peptides that promote cell penetration, such as PP75, or aptamers that enable the targeting of membrane receptors, such as AS1411. Finally, N-acetyl galactosamine (GalNAc) can be bioconjugated to promote the endocytosis of ASOs by liver cells. ASOs can be encapsulated by nanoparticles of different types: cationic polymers, inorganic particles, or lipids. They all aim to improve the stability of encapsulated ASOs, facilitate their distribution, and promote their internalization. Progress in the field of their galenic formulation is still ongoing [20,21,170]. Advances in the genetics and pathophysiology of diseases have allowed the rise of ASOs as a new therapeutic modality, making it possible to degrade or modulate the expression of defective RNAs as soon as their sequences are known. Successive advancements have led to the development of three generations of ASOs, each offering distinct advantages and disadvantages regarding physicochemical properties, pharmacokinetics, and pharmacodynamics. Their strong attractiveness compared to other therapeutic modalities lies in (i) their simple and economical synthesis, (ii) their flexible optimization, and (iii) their ability to engage targets considered non-actionable. As a result, ASOs have already proven themselves in different indications by obtaining European MAs in neurology for spinal muscular atrophy (SMA) and in metabolic disorders for human transthyretin amyloidosis (hATTR) and familial hyperchylomicronemia syndrome (FCS). The effectiveness of ASOs on monogenic diseases is impressive with a significant improvement in clinical signs. These first authorizations have made it possible to validate antisense technology as an interesting and viable therapeutic strategy for treating certain pathologies. The use of ASOs in oncology is gaining momentum, with the recent approval of imetelstat and many early-stage clinical trials showing encouraging results on key targets involved in tumor development and resistance to chemotherapy [16]. However, significant efforts are still required to consolidate data on the preclinical efficacy of ASOs and to generate robust clinical efficacy data. The success of imetelstat, a conjugated ASO, highlights the importance of meticulous design optimization. As a monotherapy, imetelstat demonstrates the potential effectiveness of ASOs even in multifactorial diseases like cancer. While targeting a single gene, as is often the case with ASOs, may not always be the most effective approach, combination strategies could offer a promising path to achieving therapeutic efficacy [171].

## Figures and Tables

**Table 1:** Summary of the most investigated chemical modifications per generation with their pharmacological properties. The first generation is modified in the phosphodiester bond by a phosphorothioate function (ASO-PS). The second generation is modified in the ribose by a methyl group (ASO-2′-O-Me) or by a methoxyethyl group (ASO-2′-MOE). This generation also comprised the gapmer structure where ASOs are modified in their 5′ and 3′ extremities with unmodified DNA molecules in the middle. The third generation is modified in its entire structure with a locked nucleic acid (ASO-LNA), phosphorodiamidate morpholino (ASO-PMO), or peptide nucleic acid (ASO-PNA).

Generation	Chemical Modifications	Improved Properties	Disadvantages	Mechanism of Action	Pharmacokinetic
First	PS	Enzymatic stability	Toxicity	RNase H degradation	Absorption Mostly parenteral routes Distribution High plasma protein binding, a large volume of distribution, and renal, hepatic, and lymphatic accumulation Metabolism Exonucleases and endonucleases Excretion As metabolites Within a few days
Second	2′-O-Me	Target binding affinity, immunogenicity, tolerability	Don’t activate RNase H	Translation inhibition, Splicing modulation + RNase H (gapmers)	Absorption Mostly parenteral routes Distribution High plasma protein binding, alarge volume of distribution, and renal, hepatic, and lymphatic accumulation Metabolism Endonuclease Excretion As metabolites Within a few weeks
	2′-MOE				
	LNA	Target binding affinity, enzymatic stability, tolerability, cellular permeability	Non-specific hybridization	Translation inhibition, Splicing modulation + RNase H (gapmers)	Absorption Mostly parenteral routes Distribution Low plasma protein binding, renal and hepatic accumulation Metabolism None Excretion Mostly intact Within hours
	PNA	Target binding affinity, hybridization rate, enzymatic stability, tolerability	Low solubility, low cellular permeability, don’t activate RNase H	Translation inhibition, Splicing modulation	
	PMO	Aqueous solubility, tolerability	Low target binding affinity, don’t activate RNase H		

## Data Availability

Not applicable.

## References

[1] Cooper T.A., Wan L., Dreyfuss G. (2009). RNA and Disease. Cell.

[2] Crooke S.T., Witztum J.L., Bennett C.F., Baker B.F. (2018). RNA-Targeted Therapeutics. Cell Metab..

[3] Poltronieri P., Sun B., Mallardo M. (2015). RNA Viruses: RNA Roles in Pathogenesis, Coreplication and Viral Load. Curr. Genom..

[4] Stephenson M.L., Zamecnik P.C. (1978). Inhibition of Rous Sarcoma Viral RNA Translation by a Specific Oligodeoxyribonucleotide. Proc. Natl. Acad. Sci. USA.

[5] Zamecnik P.C., Stephenson M.L. (1978). Inhibition of Rous Sarcoma Virus Replication and Cell Transformation by a Specific Oligodeoxynucleotide. Proc. Natl. Acad. Sci. USA.

[6] Dias N., Stein C.A. (2002). Antisense Oligonucleotides: Basic Concepts and Mechanisms. Mol. Cancer Ther..

[7] Han Y., Gao S., Muegge K., Zhang W., Zhou B. (2015). Advanced Applications of RNA Sequencing and Challenges. Bioinform. Biol. Insights.

[8] Sanghvi Y.S., Agrawal S., Gait M.J. (2019). CHAPTER 19. Large-Scale Automated Synthesis of Therapeutic Oligonucleotides: A Status Update. Drug Discovery.

[9] Lindow M., Vornlocher H.-P., Riley D., Kornbrust D.J., Burchard J., Whiteley L.O., Kamens J., Thompson J.D., Nochur S., Younis H. (2012). Assessing Unintended Hybridization-Induced Biological Effects of Oligonucleotides. Nat. Biotechnol..

[10] Yoshida T., Naito Y., Yasuhara H., Sasaki K., Kawaji H., Kawai J., Naito M., Okuda H., Obika S., Inoue T. (2019). Evaluation of Off-target Effects of Gapmer Antisense Oligonucleotides Using Human Cells. Genes Cells.

[11] Agrawal S. (1996). Antisense Oligonucleotides: Towards Clinical Trials. Trends Biotechnol..

[12] Bahal R. (2020). Antisense Oligonucleotides: An Emerging Area in Drug Discovery and Development. J. Clin. Med..

[13] Frazier K.S. (2015). Antisense Oligonucleotide Therapies: The Promise and the Challenges from a Toxicologic Pathologist’s Perspective. Toxicol. Pathol..

[14] Beer T.M., Hotte S.J., Saad F., Alekseev B., Matveev V., Fléchon A., Gravis G., Joly F., Chi K.N., Malik Z. (2017). Custirsen (OGX-011) Combined with Cabazitaxel and Prednisone versus Cabazitaxel and Prednisone Alone in Patients with Metastatic Castration-Resistant Prostate Cancer Previously Treated with Docetaxel (AFFINITY): A Randomised, Open-Label, International, Phase 3 Trial. Lancet Oncol..

[15] Stahel R.A., Zangemeister-Wittke U. (2003). Antisense Oligonucleotides for Cancer Therapy—An Overview. Lung Cancer.

[16] Xiong H., Veedu R.N., Diermeier S.D. (2021). Recent Advances in Oligonucleotide Therapeutics in Oncology. Int. J. Mol. Sci..

[17] Bennett C.F. (2019). Therapeutic Antisense Oligonucleotides Are Coming of Age. Annu. Rev. Med..

[18] Bonetta L. (2009). RNA-Based Therapeutics: Ready for Delivery?. Cell.

[19] Crooke S.T. (2007). Antisense Drug Technology Principles, Strategies, and Applications.

[20] Gagliardi M., Ashizawa A.T. (2021). The Challenges and Strategies of Antisense Oligonucleotide Drug Delivery. Biomedicines.

[21] Juliano R.L. (2016). The Delivery of Therapeutic Oligonucleotides. Nucleic Acids Res..

[22] Geary R.S., Norris D., Yu R., Bennett C.F. (2015). Pharmacokinetics, Biodistribution and Cell Uptake of Antisense Oligonucleotides. Adv. Drug Deliv. Rev..

[23] Watts J.K., Corey D.R. (2012). Silencing Disease Genes in the Laboratory and the Clinic. J. Pathol..

[24] Khvorova A., Watts J.K. (2017). The Chemical Evolution of Oligonucleotide Therapies of Clinical Utility. Nat. Biotechnol..

[25] Ammala D. (2018). Targeted Delivery of Antisense Oligonucleotides to Pancreatic β-Cells. Sci. Adv..

[26] Chan J.H., Lim S., Wong W.F. (2006). Antisense oligonucleotides: From design to therapeutic application. Clin. Exp. Pharmacol. Physiol..

[27] Vickers T.A. (2000). Effects of RNA Secondary Structure on Cellular Antisense Activity. Nucleic Acids Res..

[28] Sato K., Akiyama M., Sakakibara Y. (2021). RNA Secondary Structure Prediction Using Deep Learning with Thermodynamic Integration. Nat. Commun..

[29] Shuan-Ping Y., San-Tai S., Zhong-Ming T., Hai-Feng S. (2003). Optimization of Antisense Drug Design against Conservative Local Motif in Simulant Secondary Structures of HER-2 mRNA and QSAR Analysis. Acta Pharmacol. Sin..

[30] Zhang H.-Y. (2003). mRNA Accessible Site Tagging (MAST): A Novel High Throughput Method for Selecting Effective Antisense Oligonucleotides. Nucleic Acids Res..

[31] Matveeva O.V. (2000). Identification of Sequence Motifs in Oligonucleotides Whose Presence Is Correlated with Antisense Activity. Nucleic Acids Res..

[32] Bo X., Wang S. (2005). TargetFinder: A Software for Antisense Oligonucleotide Target Site Selection Based on MAST and Secondary Structures of Target mRNA. Bioinformatics.

[33] Mathews D.H., Turner D.H. (2006). Prediction of RNA Secondary Structure by Free Energy Minimization. Curr. Opin. Struct. Biol..

[34] Mathews D.H., Burkard M.E., Freier S.M., Wyatt J.R., Turner D.H. (1999). Predicting Oligonucleotide Affinity to Nucleic Acid Targets. RNA.

[35] Quemener A.M., Bachelot L., Forestier A., Donnou-Fournet E., Gilot D., Galibert M. (2020). The Powerful World of Antisense Oligonucleotides: From Bench to Bedside. Wiley Interdiscip. Rev. RNA.

[36] Deleavey G.F., Damha M.J. (2012). Designing Chemically Modified Oligonucleotides for Targeted Gene Silencing. Chem. Biol..

[37] Smith C.I.E., Zain R. (2019). Therapeutic Oligonucleotides: State of the Art. Annu. Rev. Pharmacol. Toxicol..

[38] Bennett C.F., Swayze E.E. (2010). RNA Targeting Therapeutics: Molecular Mechanisms of Antisense Oligonucleotides as a Therapeutic Platform. Annu. Rev. Pharmacol. Toxicol..

[39] Mansoor M., Melendez A.J. (2008). Advances in Antisense Oligonucleotide Development for Target Identification, Validation, and as Novel Therapeutics. Gene Regul. Syst. Biol..

[40] Agrawal S., Jiang Z., Zhao Q., Shaw D., Cai Q., Roskey A., Channavajjala L., Saxinger C., Zhang R. (1997). Mixed-Backbone Oligonucleotides as Second Generation Antisense Oligonucleotides: In Vitro and in Vivo Studies. Proc. Natl. Acad. Sci. USA.

[41] Monia B.P., Lesnik E.A., Gonzalez C., Lima W.F., McGee D., Guinosso C.J., Kawasaki A.M., Cook P.D., Freier S.M. (1993). Evaluation of 2′-Modified Oligonucleotides Containing 2‘-Deoxy Gaps as Antisense Inhibitors of Gene Expression. J. Biol. Chem..

[42] Braasch D.A., Corey D.R. (2001). Locked Nucleic Acid (LNA): ¢ne-Tuning the Recognition of DNA and RNA. Chem. Biol..

[43] Heasman J. (2002). Morpholino Oligos: Making Sense of Antisense?. Dev. Biol..

[44] Braasch D.A., Corey D.R. (2002). Novel Antisense and Peptide Nucleic Acid Strategies for Controlling Gene Expression. Biochemistry.

[45] Crooke S.T. (2017). Molecular Mechanisms of Antisense Oligonucleotides. Nucleic Acid Ther..

[46] Gad S.C. (2010). Pharmaceutical Sciences Encyclopedia: Drug Discovery, Development, and Manufacturing.

[47] Zhang Y.C., Taylor M.M., Samson W.K., Phillips M.I. (2005). Antisense Inhibition: Oligonucleotides, Ribozymes, and siRNAs. Methods Mol. Med..

[48] Cerritelli S.M., Crouch R.J. (2009). Ribonuclease H: The Enzymes in Eukaryotes: Ribonucleases H of Eukaryotes. FEBS J..

[49] Wu H., Lima W.F., Zhang H., Fan A., Sun H., Crooke S.T. (2004). Determination of the Role of the Human RNase H1 in the Pharmacology of DNA-like Antisense Drugs. J. Biol. Chem..

[50] Witztum J.L., Gaudet D., Freedman S.D., Alexander V.J., Digenio A., Williams K.R., Yang Q., Hughes S.G., Geary R.S., Arca M. (2019). Volanesorsen and Triglyceride Levels in Familial Chylomicronemia Syndrome. N. Engl. J. Med..

[51] Di Fusco D., Dinallo V., Marafini I., Figliuzzi M.M., Romano B., Monteleone G. (2019). Antisense Oligonucleotide: Basic Concepts and Therapeutic Application in Inflammatory Bowel Disease. Front. Pharmacol..

[52] Kiełpiński Ł.J., Hagedorn P.H., Lindow M., Vinther J. (2017). RNase H Sequence Preferences Influence Antisense Oligonucleotide Efficiency. Nucleic Acids Res..

[53] Nilsson J.A., Cleveland J.L. (2003). Myc Pathways Provoking Cell Suicide and Cancer. Oncogene.

[54] Devi G.R., Beer T.M., Corless C.L., Arora V., Weller D.L., Iversen P.L. (2005). *In Vivo* Bioavailability and Pharmacokinetics of a *c-MYC* Antisense Phosphorodiamidate Morpholino Oligomer, AVI-4126, in Solid Tumors. Clin. Cancer Res..

[55] Bauman J., Jearawiriyapaisarn N., Kole R. (2009). Therapeutic Potential of Splice-Switching Oligonucleotides. Oligonucleotides.

[56] Aartsma-Rus A., De Waele L., Houwen-Opstal S., Kirschner J., Krom Y.D., Mercuri E., Niks E.H., Straub V., van Duyvenvoorde H.A., Vroom E. (2023). The Dilemma of Choice for Duchenne Patients Eligible for Exon 51 Skipping The European Experience. J. Neuromuscul. Dis..

[57] Havens M.A., Duelli D.M., Hastings M.L. (2013). Targeting RNA Splicing for Disease Therapy: RNA Splicing for Disease Therapy. Wiley Interdiscip. Rev. RNA.

[58] Chiriboga C.A. (2017). Nusinersen for the Treatment of Spinal Muscular Atrophy. Expert Rev. Neurother..

[59] Goemans N.M., Tulinius M., van den Akker J.T., Burm B.E., Ekhart P.F., Heuvelmans N., Holling T., Janson A.A., Platenburg G.J., Sipkens J.A. (2011). Systemic Administration of PRO051 in Duchenne’s Muscular Dystrophy. N. Engl. J. Med..

[60] Bogdahn U., Hau P., Stockhammer G., Venkataramana N.K., Mahapatra A.K., Suri A., Balasubramaniam A., Nair S., Oliushine V., Parfenov V. (2011). Targeted Therapy for High-Grade Glioma with the TGF- 2 Inhibitor Trabedersen: Results of a Randomized and Controlled Phase IIb Study. Neuro-Oncology.

[61] Miller T.M., Pestronk A., David W., Rothstein J., Simpson E., Appel S.H., Andres P.L., Mahoney K., Allred P., Alexander K. (2013). An Antisense Oligonucleotide against SOD1 Delivered Intrathecally for Patients with SOD1 Familial Amyotrophic Lateral Sclerosis: A Phase 1, Randomised, First-in-Man Study. Lancet Neurol..

[62] de Smet M.D., Meenken C., van den Horn G.J. (1999). Fomivirsen–A Phosphorothioate Oligonucleotide for the Treatment of CMV Retinitis. Ocul. Immunol. Inflamm..

[63] Lightfoot H., Schneider A., Hall J., Ferrari N., Seguin R. (2018). Pharmacokinetics and Pharmacodynamics of Antisense Oligonucleotides. Oligonucleotide-Based Drugs and Therapeutics.

[64] Yu R.Z., Kim T.-W., Hong A., Watanabe T.A., Gaus H.J., Geary R.S. (2007). Cross-Species Pharmacokinetic Comparison from Mouse to Man of a Second-Generation Antisense Oligonucleotide, ISIS 301012, Targeting Human Apolipoprotein B-100. Drug Metab. Dispos..

[65] Geary R.S. (2009). Antisense Oligonucleotide Pharmacokinetics and Metabolism. Expert Opin. Drug Metab. Toxicol..

[66] Amantana A., Iversen P. (2005). Pharmacokinetics and Biodistribution of Phosphorodiamidate Morpholino Antisense Oligomers. Curr. Opin. Pharmacol..

[67] Dirin M., Winkler J. (2013). Influence of Diverse Chemical Modifications on the ADME Characteristics and Toxicology of Antisense Oligonucleotides. Expert Opin. Biol. Ther..

[68] McMahon B.M., Mays D., Lipsky J., Stewart J.A., Fauq A., Richelson E. (2002). Pharmacokinetics and Tissue Distribution of a Peptide Nucleic Acid After Intravenous Administration. Antisense Nucleic Acid Drug Dev..

[69] Lendvai G., Velikyan I., Estrada S., Eriksson B., Långström B., Bergström M. (2008). Biodistribution of 68Ga-Labeled LNA–DNA Mixmer Antisense Oligonucleotides for Rat Chromogranin-A. Oligonucleotides.

[70] Crooke S.T., Wang S., Vickers T.A., Shen W., Liang X. (2017). Cellular Uptake and Trafficking of Antisense Oligonucleotides. Nat. Biotechnol..

[71] Yu C., Brussaard A.B., Yang X., Listerud M., Role L.W. (1993). Uptake of Antisense Oligonucleotides and Functional Block of Acetylcholine Receptor Subunit Gene Expression in Primary Embryonic Neurons. Dev. Genet..

[72] Koller E., Vincent T.M., Chappell A., De S., Manoharan M., Bennett C.F. (2011). Mechanisms of Single-Stranded Phosphorothioate Modified Antisense Oligonucleotide Accumulation in Hepatocytes. Nucleic Acids Res..

[73] Alam M.R., Dixit V., Kang H., Li Z.-B., Chen X., Trejo J., Fisher M., Juliano R.L. (2008). Intracellular Delivery of an Anionic Antisense Oligonucleotide via Receptor-Mediated Endocytosis. Nucleic Acids Res..

[74] Ming X., Alam M.R., Fisher M., Yan Y., Chen X., Juliano R.L. (2010). Intracellular Delivery of an Antisense Oligonucleotide via Endocytosis of a G Protein-Coupled Receptor. Nucleic Acids Res..

[75] Miller C.M., Donner A.J., Blank E.E., Egger A.W., Kellar B.M., Østergaard M.E., Seth P.P., Harris E.N. (2016). Stabilin-1 and Stabilin-2 Are Specific Receptors for the Cellular Internalization of Phosphorothioate-Modified Antisense Oligonucleotides (ASOs) in the Liver. Nucleic Acids Res..

[76] Juliano R.L., Ming X., Carver K., Laing B. (2014). Cellular Uptake and Intracellular Trafficking of Oligonucleotides: Implications for Oligonucleotide Pharmacology. Nucleic Acid Ther..

[77] Doherty G.J., McMahon H.T. (2009). Mechanisms of Endocytosis. Annu. Rev. Biochem..

[78] Juliano R.L., Ming X., Nakagawa O. (2012). Cellular Uptake and Intracellular Trafficking of Antisense and siRNA Oligonucleotides. Bioconjug. Chem..

[79] Juliano R.L., Carver K., Cao C., Ming X. (2013). Receptors, Endocytosis, and Trafficking: The Biological Basis of Targeted Delivery of Antisense and siRNA Oligonucleotides. J. Drug Target..

[80] Linnane E., Davey P., Zhang P., Puri S., Edbrooke M., Chiarparin E., Revenko A.S., Macleod A.R., Norman J.C., Ross S.J. (2019). Differential Uptake, Kinetics and Mechanisms of Intracellular Trafficking of next-Generation Antisense Oligonucleotides across Human Cancer Cell Lines. Nucleic Acids Res..

[81] Geary R.S., Wancewicz E., Matson J., Pearce M., Siwkowski A., Swayze E., Bennett F. (2009). Effect of Dose and Plasma Concentration on Liver Uptake and Pharmacologic Activity of a 2′-Methoxyethyl Modified Chimeric Antisense Oligonucleotide Targeting PTEN. Biochem. Pharmacol..

[82] Juliano R.L., Carver K. (2015). Cellular Uptake and Intracellular Trafficking of Oligonucleotides. Adv. Drug Deliv. Rev..

[83] Goldenring J.R. (2015). Recycling Endosomes. Curr. Opin. Cell Biol..

[84] Huotari J., Helenius A. (2011). Endosome Maturation: Endosome Maturation. EMBO J..

[85] Hanson P.I., Cashikar A. (2012). Multivesicular Body Morphogenesis. Annu. Rev. Cell Dev. Biol..

[86] Luzio J.P., Pryor P.R., Bright N.A. (2007). Lysosomes: Fusion and Function. Nat. Rev. Mol. Cell Biol..

[87] Varkouhi A.K., Scholte M., Storm G., Haisma H.J. (2011). Endosomal Escape Pathways for Delivery of Biologicals. J. Control. Release.

[88] Marchese A., Paing M.M., Temple B.R.S., Trejo J. (2008). G Protein–Coupled Receptor Sorting to Endosomes and Lysosomes. Annu. Rev. Pharmacol. Toxicol..

[89] Kapustin A.N., Davey P., Longmire D., Matthews C., Linnane E., Rustogi N., Stavrou M., Devine P.W.A., Bond N.J., Hanson L. (2021). Antisense Oligonucleotide Activity in Tumour Cells Is Influenced by Intracellular LBPA Distribution and Extracellular Vesicle Recycling. Commun. Biol..

[90] Burel S.A., Hart C.E., Cauntay P., Hsiao J., Machemer T., Katz M., Watt A., Bui H., Younis H., Sabripour M. (2016). Hepatotoxicity of High Affinity Gapmer Antisense Oligonucleotides Is Mediated by RNase H1 Dependent Promiscuous Reduction of Very Long Pre-mRNA Transcripts. Nucleic Acids Res..

[91] Fragall C.T., Adams A.M., Johnsen R.D., Kole R., Fletcher S., Wilton S.D. (2011). Mismatched Single Stranded Antisense Oligonucleotides Can Induce Efficient Dystrophin Splice Switching. BMC Med. Genet..

[92] Monia B.P., Johnston J.F., Geiger T., Muller M., Fabbro D. (1996). Antitumor Activity of a Phosphorothioate Antisense Oligodeoxynucleotide Targeted against C-Raf Kinase. Nat. Med..

[93] Jackson A.L., Burchard J., Schelter J., Chau B.N., Cleary M., Lim L., Linsley P.S. (2006). Widespread siRNA “off-Target” Transcript Silencing Mediated by Seed Region Sequence Complementarity. RNA.

[94] Agrawal S., Kandimalla E.R. (2004). Antisense and siRNA as Agonists of Toll-like Receptors. Nat. Biotechnol..

[95] Senn J.J., Burel S., Henry S.P. (2005). Non-CpG-Containing Antisense 2′-Methoxyethyl Oligonucleotides Activate a Proinflammatory Response Independent of Toll-Like Receptor 9 or Myeloid Differentiation Factor 88. J. Pharmacol. Exp. Ther..

[96] Choi S.-S., Chung E., Jung Y.-J. (2010). Newly Identified CpG ODNs, M5-30 and M6-395, Stimulate Mouse Immune Cells to Secrete TNF-α and Enhance Th1-Mediated Immunity. J. Microbiol..

[97] Henry S.P., Beattie G., Yeh G., Chappel A., Giclas P., Mortari A., Jagels M.A., Kornbrust D.J., Levin A.A. (2002). Complement Activation Is Responsible for Acute Toxicities in Rhesus Monkeys Treated with a Phosphorothioate Oligodeoxynucleotide. Int. Immunopharmacol..

[98] FDA (2012). EMDAC Clinical Briefing Document, NDA 203568, Mipomersen Sodium Injection 200 mg/mL 2012.

[99] Burdick A.D., Sciabola S., Mantena S.R., Hollingshead B.D., Stanton R., Warneke J.A., Zeng M., Martsen E., Medvedev A., Makarov S.S. (2014). Sequence Motifs Associated with Hepatotoxicity of Locked Nucleic Acid—Modified Antisense Oligonucleotides. Nucleic Acids Res..

[100] Kakiuchi-Kiyota S., Koza-Taylor P.H., Mantena S.R., Nelms L.F., Enayetallah A.E., Hollingshead B.D., Burdick A.D., Reed L.A., Warneke J.A., Whiteley L.O. (2014). Comparison of Hepatic Transcription Profiles of Locked Ribonucleic Acid Antisense Oligonucleotides: Evidence of Distinct Pathways Contributing to Non-Target Mediated Toxicity in Mice. Toxicol. Sci..

[101] Hagedorn P.H., Yakimov V., Ottosen S., Kammler S., Nielsen N.F., Høg A.M., Hedtjärn M., Meldgaard M., Møller M.R., Ørum H. (2013). Hepatotoxic Potential of Therapeutic Oligonucleotides Can Be Predicted from Their Sequence and Modification Pattern. Nucleic Acid Ther..

[102] (2008). Henry Toxicologic Properties of 20-Methoxyethyl Chimeric Antisense Inhibitors in Animals and Man. Antisense Drug Technology: Principles, Strategies and Applications.

[103] Levin A.A., Henry S.P. (2010). Toxicology of Oligonucleotide Therapeutics and Understanding the Relevance of the Toxicities. Pharmaceutical Sciences Encyclopedia.

[104] Jaax M.E., Krauel K., Marschall T., Brandt S., Gansler J., Fürll B., Appel B., Fischer S., Block S., Helm C.A. (2013). Complex Formation with Nucleic Acids and Aptamers Alters the Antigenic Properties of Platelet Factor 4. Blood.

[105] Henry S.P., Geary R.S., Yu R., Levin A.A. (2001). Drug Properties of Second-Generation Antisense Oligonucleotides: How Do They Measure up to Their Predecessors?. Curr. Opin. Investig. Drugs.

[106] Cieślik M., Chinnaiyan A.M. (2018). Cancer Transcriptome Profiling at the Juncture of Clinical Translation. Nat. Rev. Genet..

[107] Gleave M.E., Monia B.P. (2005). Antisense Therapy for Cancer. Nat. Rev. Cancer.

[108] Paccosi E., Costantino M., Balzerano A., Filippi S., Brancorsini S., Proietti-De-Santis L. (2021). Neuroblastoma Cells Depend on CSB for Faithful Execution of Cytokinesis and Survival. Int. J. Mol. Sci..

[109] Filippi S., Paccosi E., Balzerano A., Ferretti M., Poli G., Taborri J., Brancorsini S., Proietti-De-Santis L. (2022). CSA Antisense Targeting Enhances Anticancer Drug Sensitivity in Breast Cancer Cells, Including the Triple-Negative Subtype. Cancers.

[110] Bartolucci D., Pession A., Hrelia P., Tonelli R. (2022). Precision Anti-Cancer Medicines by Oligonucleotide Therapeutics in Clinical Research Targeting Undruggable Proteins and Non-Coding RNAs. Pharmaceutics.

[111] Harris M.H., Thompson C.B. (2000). The Role of the Bcl-2 Family in the Regulation of Outer Mitochondrial Membrane Permeability. Cell Death Differ..

[112] Campbell K.J., Tait S.W.G. (2018). Targeting BCL-2 Regulated Apoptosis in Cancer. Open Biol..

[113] (2007). Genta Oblimersen: Augmerosen, BCL-2 Antisense Oligonucleotide—Genta, G 3139, GC 3139, Oblimersen Sodium. Drugs R D.

[114] Waters J.S., Webb A., Cunningham D., Clarke P.A., Raynaud F., di Stefano F., Cotter F.E. (2000). Phase I Clinical and Pharmacokinetic Study of Bcl-2 Antisense Oligonucleotide Therapy in Patients With Non-Hodgkin’s Lymphoma. J. Clin. Oncol..

[115] Pham T.-H., Park H.-M., Kim J., Hong J.T., Yoon D.-Y. (2020). STAT3 and P53: Dual Target for Cancer Therapy. Biomedicines.

[116] Kamran M.Z., Patil P., Gude R.P. (2013). Role of STAT3 in Cancer Metastasis and Translational Advances. BioMed Res. Int..

[117] Furqan M., Akinleye A., Mukhi N., Mittal V., Chen Y., Liu D. (2013). STAT Inhibitors for Cancer Therapy. J. Hematol. Oncol..

[118] Xu H., Tong X., Mugundu G., Scott M.L., Cook C., Arfvidsson C., Pease E., Zhou D., Lyne P., Al-Huniti N. (2019). Population Pharmacokinetic Analysis of Danvatirsen Supporting Flat Dosing Switch. J. Pharmacokinet. Pharmacodyn..

[119] Reilley M.J., McCoon P., Cook C., Lyne P., Kurzrock R., Kim Y., Woessner R., Younes A., Nemunaitis J., Fowler N. (2018). STAT3 Antisense Oligonucleotide AZD9150 in a Subset of Patients with Heavily Pretreated Lymphoma: Results of a Phase 1b Trial. J. Immunother. Cancer.

[120] Garg R., Benedetti L.G., Abera M.B., Wang H., Abba M., Kazanietz M.G. (2014). Protein Kinase C and Cancer: What We Know and What We Do Not. Oncogene.

[121] Lahn M., Köhler G., Sundell K., Su C., Li S., Paterson B.M., Bumol T.F. (2004). Protein Kinase C Alpha Expression in Breast and Ovarian Cancer. Oncology.

[122] Roychowdhury D., Lahn M. (2003). Antisense Therapy Directed to Protein Kinase C-Alpha (Affinitak, LY900003/ISIS 3521): Potential Role in Breast Cancer. Semin. Oncol..

[123] Villalona-Calero M.A., Ritch P., Figueroa J.A., Otterson G.A., Belt R., Dow E., George S., Leonardo J., McCachren S., Miller G.L. (2004). A Phase I/II Study of LY900003, an Antisense Inhibitor of Protein Kinase C-α, in Combination with Cisplatin and Gemcitabine in Patients with Advanced Non–Small Cell Lung Cancer. Clin. Cancer Res..

[124] Paz-Ares L., Douillard J.-Y., Koralewski P., Manegold C., Smit E.F., Reyes J.M., Chang G.-C., John W.J., Peterson P.M., Obasaju C.K. (2006). Phase III Study of Gemcitabine and Cisplatin With or Without Aprinocarsen, a Protein Kinase C-Alpha Antisense Oligonucleotide, in Patients With Advanced-Stage Non–Small-Cell Lung Cancer. J. Clin. Oncol..

[125] Kelland L. (2006). Discontinued Drugs in 2005: Oncology Drugs. Expert Opin. Investig. Drugs.

[126] Trougakos I.P., Pawelec G., Tzavelas C., Ntouroupi T., Gonos E.S. (2006). Clusterin/Apolipoprotein J up-Regulation after Zinc Exposure, Replicative Senescence or Differentiation of Human Haematopoietic Cells. Biogerontology.

[127] Peng M., Deng J., Zhou S., Tao T., Su Q., Xue Y., Yang X. (2019). The Role of Clusterin in Cancer Metastasis. Cancer Manag. Res..

[128] Wang Y., Wang X., Zhao H., Liang B., Du Q. (2012). Clusterin Confers Resistance to TNF-Alpha-Induced Apoptosis in Breast Cancer Cells through NF-kappaB Activation and Bcl-2 Overexpression. J. Chemother..

[129] Chi K.N., Zoubeidi A., Gleave M.E. (2008). Custirsen (OGX-011): A Second-Generation Antisense Inhibitor of Clusterin for the Treatment of Cancer. Expert Opin. Investig. Drugs.

[130] Chi K.N., Higano C.S., Blumenstein B., Ferrero J.-M., Reeves J., Feyerabend S., Gravis G., Merseburger A.S., Stenzl A., Bergman A.M. (2017). Custirsen in Combination with Docetaxel and Prednisone for Patients with Metastatic Castration-Resistant Prostate Cancer (SYNERGY Trial): A Phase 3, Multicentre, Open-Label, Randomised Trial. Lancet Oncol..

[131] Eckelman B.P., Salvesen G.S., Scott F.L. (2006). Human Inhibitor of Apoptosis Proteins: Why XIAP Is the Black Sheep of the Family. EMBO Rep..

[132] Abbas R., Larisch S. (2020). Targeting XIAP for Promoting Cancer Cell Death—The Story of ARTS and SMAC. Cells.

[133] Lee F.A.S., Zee B.C.-Y., Cheung F.Y., Kwong P., Chiang C.L., Leung K.C., Siu S.W.K., Lee C., Lai M., Kwok C. (2016). Randomized Phase II Study of the X-Linked Inhibitor of Apoptosis (XIAP) Antisense AEG35156 in Combination With Sorafenib in Patients With Advanced Hepatocellular Carcinoma (HCC). Am. J. Clin. Oncol..

[134] Herbert B.-S., Gellert G.C., Hochreiter A., Pongracz K., Wright W.E., Zielinska D., Chin A.C., Harley C.B., Shay J.W., Gryaznov S.M. (2005). Lipid Modification of GRN163, an N3′ → P5′ Thio-Phosphoramidate Oligonucleotide, Enhances the Potency of Telomerase Inhibition. Oncogene.

[135] Asai A., Oshima Y., Yamamoto Y., Uochi T., Kusaka H., Akinaga S., Yamashita Y., Pongracz K., Pruzan R., Wunder E. (2003). A Novel Telomerase Template Antagonist (GRN163) as a Potential Anticancer Agent. Cancer Res..

[136] Jafri M.A., Ansari S.A., Alqahtani M.H., Shay J.W. (2016). Roles of Telomeres and Telomerase in Cancer, and Advances in Telomerase-Targeted Therapies. Genome Med..

[137] Platzbecker U., Santini V., Fenaux P., Sekeres M.A., Savona M.R., Madanat Y.F., Díez-Campelo M., Valcárcel D., Illmer T., Jonášová A. (2024). Imetelstat in Patients with Lower-Risk Myelodysplastic Syndromes Who Have Relapsed or Are Refractory to Erythropoiesis-Stimulating Agents (IMerge): A Multinational, Randomised, Double-Blind, Placebo-Controlled, Phase 3 Trial. Lancet.

[138] Caruso G., Caffo M. (2014). Antisense Oligonucleotides in the Treatment of Cerebral Gliomas. Review of Concerning Patents. Recent Pat. CNS Drug Discov..

[139] Ostrom Q.T., Gittleman H., Liao P., Rouse C., Chen Y., Dowling J., Wolinsky Y., Kruchko C., Barnholtz-Sloan J. (2014). CBTRUS Statistical Report: Primary Brain and Central Nervous System Tumors Diagnosed in the United States in 2007–2011. Neuro-Oncology.

[140] Yuile P., Dent O., Cook R., Biggs M., Little N. (2006). Survival of Glioblastoma Patients Related to Presenting Symptoms, Brain Site and Treatment Variables. J. Clin. Neurosci..

[141] Sanai N., Polley M.-Y., McDermott M.W., Parsa A.T., Berger M.S. (2011). An Extent of Resection Threshold for Newly Diagnosed Glioblastomas: Clinical Article. J. Neurosurg..

[142] Wirsching H.-G., Galanis E., Weller M. (2016). Glioblastoma. Handbook of Clinical Neurology.

[143] Weller M., van den Bent M., Hopkins K., Tonn J.C., Stupp R., Falini A., Cohen-Jonathan-Moyal E., Frappaz D., Henriksson R., Balana C. (2014). EANO Guideline for the Diagnosis and Treatment of Anaplastic Gliomas and Glioblastoma. Lancet Oncol..

[144] Dean N.M., Mckay R. (1994). Inhibition of Protein Kinase C-a Expression in Mice after Systemic Administration of Phosphorothioate Antisense Oligodeoxynucleotides. Proc. Natl. Acad. Sci. USA.

[145] Yazaki T., Ahmad S., Chahlavi A., Zylber-Katz E., Dean N.M., Rabkin S.D., Martuza R.L., Glazer R.I. (1996). Treatment of Glioblastoma U-87 by Systemic Administration of an Antisense Protein Kinase C-Alpha Phosphorothioate Oligodeoxynucleotide. Mol. Pharmacol..

[146] Nemunaitis J., Holmlund J.T., Kraynak M., Richards D., Bruce J., Ognoskie N., Kwoh T.J., Geary R., Dorr A., Von Hoff D. (1999). Phase I Evaluation of ISIS 3521, an Antisense Oligodeoxynucleotide to Protein Kinase C-Alpha, in Patients With Advanced Cancer. J. Clin. Oncol..

[147] Grossman S.A., Alavi J.B., Supko J.G., Carson K.A., Priet R., Dorr F.A., Grundy J.S., Holmlund J.T. (2005). Efficacy and Toxicity of the Antisense Oligonucleotide Aprinocarsendirected against Protein Kinase C-α Delivered as a 21-Day Continuousintravenous Infusion in Patients with Recurrent High-Grade Astrocytomas. Neuro-Oncology.

[148] Schlingensiepen K., Schlingensiepen R., Steinbrecher A., Hau P., Bogdahn U., Fischerblass B., Jachimczak P. (2006). Targeted Tumor Therapy with the TGF-Β2 Antisense Compound AP 12009. Cytokine Growth Factor Rev..

[149] Massagué J. (1998). TGF-β Signal Transduction. Annu. Rev. Biochem..

[150] Huang T., Schor S.L., Hinck A.P. (2014). Biological Activity Differences between TGF-Β1 and TGF-Β3 Correlate with Differences in the Rigidity and Arrangement of Their Component Monomers. Biochemistry.

[151] Rahimi R.A., Leof E.B. (2007). TGF-β Signaling: A Tale of Two Responses. J. Cell. Biochem..

[152] Joseph J.V., Balasubramaniyan V., Walenkamp A., Kruyt F.A.E. (2013). TGF-β as a Therapeutic Target in High Grade Gliomas–Promises and Challenges. Biochem. Pharmacol..

[153] Schlingensiepen R., Goldbrunner M., Szyrach M.N.I., Stauder G., Jachimczak P., Bogdahn U., Schulmeyer F., Hau P., Schlingensiepen K.-H. (2005). Intracerebral and Intrathecal Infusion of the TGF-Β2-Specific Antisense Phosphorothioate Oligonucleotide AP 12009 in Rabbits and Primates: Toxicology and Safety. Oligonucleotides.

[154] Hau P., Jachimczak P., Schlingensiepen R., Schulmeyer F., Jauch T., Steinbrecher A., Brawanski A., Proescholdt M., Schlaier J., Buchroithner J. (2007). Inhibition of TGF-*β* 2 with AP 12009 in Recurrent Malignant Gliomas: From Preclinical to Phase I/II Studies. Oligonucleotides.

[155] Krichevsky A.M., Uhlmann E.J. (2019). Oligonucleotide Therapeutics as a New Class of Drugs for Malignant Brain Tumors: Targeting mRNAs, Regulatory RNAs, Mutations, Combinations, and Beyond. Neurotherapeutics.

[156] Chamberlain M.C. (2011). Convection-Enhanced Delivery of a Transforming Growth Factor-Β2 Inhibitor Trabedersen for Recurrent High-Grade Gliomas: Efficacy Real or Imagined? In Reference to Bogdahn et al. (Neuro-Oncology 2011;13:132–142). Neuro-Oncology.

[157] McNeill R.S., Vitucci M., Wu J., Miller C.R. (2015). Contemporary Murine Models in Preclinical Astrocytoma Drug Development. Neuro-Oncology.

[158] Sarkaria J.N., Hu L.S., Parney I.F., Pafundi D.H., Brinkmann D.H., Laack N.N., Giannini C., Burns T.C., Kizilbash S.H., Laramy J.K. (2018). Is the Blood–Brain Barrier Really Disrupted in All Glioblastomas? A Critical Assessment of Existing Clinical Data. Neuro-Oncology.

[159] Idbaih A., Canney M., Belin L., Desseaux C., Vignot A., Bouchoux G., Asquier N., Law-Ye B., Leclercq D., Bissery A. (2019). Safety and Feasibility of Repeated and Transient Blood-Brain Barrier Disruption by Pulsed Ultrasound in Patients with Recurrent Glioblastoma. Clin. Cancer Res. Off. J. Am. Assoc. Cancer Res..

[160] Zhao G., Huang Q., Wang F., Zhang X., Hu J., Tan Y., Huang N., Wang Z., Wang Z., Cheng Y. (2018). Targeted shRNA-Loaded Liposome Complex Combined with Focused Ultrasound for Blood Brain Barrier Disruption and Suppressing Glioma Growth. Cancer Lett..

[161] Mehta A.M., Sonabend A.M., Bruce J.N. (2017). Convection-Enhanced Delivery. Neurotherapeutics.

[162] Roberts T.C., Langer R., Wood M.J.A. (2020). Advances in Oligonucleotide Drug Delivery. Nat. Rev. Drug Discov..

[163] Wan L.-Y., Yuan W.-F., Ai W.-B., Ai Y.-W., Wang J.-J., Chu L.-Y., Zhang Y.-Q., Wu J.-F. (2019). An Exploration of Aptamer Internalization Mechanisms and Their Applications in Drug Delivery. Expert Opin. Drug Deliv..

[164] Ireson C.R., Kelland L.R. (2006). Discovery and Development of Anticancer Aptamers. Mol. Cancer Ther..

[165] Khormaee S., Choi Y., Shen M.J., Xu B., Wu H., Griffiths G.L., Chen R., Slater N.K.H., Park J.K. (2013). Endosomolytic Anionic Polymer for the Cytoplasmic Delivery of siRNAs in Localized In Vivo Applications. Adv. Funct. Mater..

[166] Salloum R., Hummel T.R., Kumar S.S., Dorris K., Li S., Lin T., Daryani V.M., Stewart C.F., Miles L., Poussaint T.Y. (2016). A Molecular Biology and Phase II Study of Imetelstat (GRN163L) in Children with Recurrent or Refractory Central Nervous System Malignancies: A Pediatric Brain Tumor Consortium Study. J. Neurooncol..

[167] Adjiri A. (2017). DNA Mutations May Not Be the Cause of Cancer. Oncol. Ther..

[168] Rudek M.A., Chau C.H., Figg W.D., McLeod H.L. (2014). Handbook of Anticancer Pharmacokinetics and Pharmacodynamics.

[169] Kilanowska A., Studzińska S. (2020). In Vivo and In Vitro Studies of Antisense Oligonucleotides—A Review. RSC Adv..

[170] Vidal L., Blagden S., Attard G., de Bono J. (2005). Making Sense of Antisense. Eur. J. Cancer.

[171] Biroccio A., Leonetti C., Zupi G. (2003). The Future of Antisense Therapy: Combination with Anticancer Treatments. Oncogene.

